# BCAA metabolism: the Achilles’ heel of ovarian cancer, polycystic ovary syndrome, and premature ovarian failure

**DOI:** 10.3389/fendo.2025.1579477

**Published:** 2025-07-04

**Authors:** Tian Zeng, Yitong Liu, Xing Tang, Runshu Fu, Qing Gao, Wenchao Zhou, Jiawen Fang, Juan Zhang, Juan Zou, Yukun Li

**Affiliations:** ^1^ Department of Assisted Reproductive Centre, The affiliated Zhuzhou Hospital Xiangya Medical College, Central South University, Zhuzhou, Hunan, China; ^2^ Hunan Province Key Laboratory of Tumor Cellular & Molecular Pathology, Cancer Research Institute, University of South China, Hengyang, Hunan, China; ^3^ Department of Pathology, Shenzhen Baoan Women’s and Children’s Hospital, Shenzhen, Guangdong, China; ^4^ Hunan Province Cooperative Innovation Center for Molecular Target New Drug Study, School of Pharmaceutical Science, Hengyang Medical School, University of South China, Hengyang, Hunan, China; ^5^ School of Nursing, Heilongjiang University of Chinese Medicine, Harbin, Heilongjiang, China

**Keywords:** branched-chain amino acids (BCAAs), human ovarian disease, metabolism, polycystic ovary syndrome (PCOS), premature ovarian failure (POF)

## Abstract

Branched-chain amino acids (BCAAs), including valine, leucine and isoleucine, are essential nutrient signals that influence mammalian animal metabolism. Many enzymes are involved in the metabolism of BCAAs, such as branched-chain amino acid transaminases (BCATs), branched-chain α-keto acid dehydrogenase (BCKDH), and BCKDH kinase (BCKDK). The aberrant expression of enzymes involved in BCAA metabolism and an imbalance in BCAA amino acid intake can lead to disordered metabolism. Aberrant BCAA metabolism can lead to several diseases, such as human ovarian disease, including ovarian cancer (OC), polycystic ovary syndrome (PCOS), and premature ovarian failure (POF), which are common gynaecological diseases. The overexpression of BCATs is found in OC, which promotes BCAA catalysis to provide a large amount of energy for tumorigenesis. However, BCKDK is overexpressed in epithelial ovarian cancer (EOC), which promotes proliferation and migration via MEK–ERK. In addition, several studies have reported that high levels of BCAAs are increased in the plasma of PCOS and POF patients. This review focuses on the role of BCAA metabolism and potential management methods for OC, PCOS and POF.

## 1 Introduction

BCAAs, such as valine, isoleucine, and leucine, are common amino acids in proteins and participate in nutrition metabolism in mammals (1). Previous studies have suggested that BCAA metabolism plays an essential role in various diseases. Increasing BCAA levels in the blood of patients with insulin resistance and obesity were first reported in the 1960s (2, 3). Recent studies have shown that BCAAs contribute to insulin resistance and suggest that BCAAs can be used as predictors for detecting insulin resistance (4, 5). The role of BCAA metabolism in cardiovascular disease and heart failure has also been noted (6). Wang et al. reported that oral BCAA intake increased cardiac BCAA levels in myocardial infarction (MI)-operated mice and exacerbated cardiac dysfunction and remodelling through the mammalian target of rapamycin (mTOR) (7). Moreover, as important amino acids in nutrient metabolism, altered BCAA metabolism has been associated with tumorigenesis (8). Recent studies have indicated that elevated plasma levels of BCAA are related to an increased risk of developing pancreatic ductal adenocarcinoma (9). However, Budhathoki et al. reported that high levels of BCAAs are negatively associated with colorectal adenoma and indicated that BCAAs may have a beneficial effect against colorectal carcinogenesis (10). Changes in amino acids may be potential biomarkers and therapeutic targets for diseases. Thus, the role of BCAA metabolism in the occurrence and development of diseases is noteworthy.

OC is the third most common gynaecologic malignancy worldwide and has a high mortality rate (11). On the basis of histopathology and molecular genetic alterations, OC can be divided into EOC (12) and nonepithelial ovarian cancer. The most common types of EOC are divided into five main types: high-grade serous (70%), clear-cell (10%), endometrioid (10%), mucinous (3%), and low-grade serous carcinomas (<5%) (13). Like most cancers, the cause of OC is unclear. The lack of clinically specific symptoms makes early diagnosis difficult, and late diagnosis is responsible for the poor survival rates of patients with cancer. It is necessary to identify early specific markers of OC (14–16). The treatment of gynaecological tumours has greatly improved with the development of modern molecular medicine (17). With the study of metabolomics, tumour metabolism has become a novel target of drug therapy (18). Zhang et al. identified eight differentially expressed metabolism-related genes that could be used as predictors in ovarian serous cystadenocarcinoma, including the PYGB, ADH1B, ADCY9, HPGDS, ENPP1, CYP2E1, FH, and NDUFA5 genes (19). However, metabolic reprogramming was initially seen as a consequence of oncogenesis. The study of metabolic enzymes revealed that metabolic reprogramming may be the cause of tumour transformation (20). Aberrant metabolism may lead to tumorigenesis. Phillips et al. reported that the trans-sulfuration enzyme cystathionine-β-synthase (CBS) and its product hydrogen sulfide (H2S) are significantly upregulated in colorectal cancers and confirmed that activation of the CBS-H2S axis facilitates colon carcinogenesis (21). Gatenby et al. suggested that increased glycolysis results in the production of lactate via lactate dehydrogenase and that the acidic microenvironment promotes tumour invasion and metastasis (22). Hence, aberrant metabolism plays an important role in tumorigenesis and progression (18, 23, 24). Exploring the role of metabolism in OC may provide help for early diagnosis in the future.

PCOS is a prevalent endocrine disease that affects reproductive-aged females and is characterized by hyperandrogenism, oligomenorrhea and amenorrhea, leading to infertility, obesity, insulin resistance, cardiovascular problems and serious health issues (25). The morphological changes in PCOS patients mainly include vast nonmaturating and atretic follicles, luteinized inner theca, thickening of the ovarian cortex, multiple tiny cystic follicular cysts and aberrant ovarian hyperplasia. Compared with that of normal ovaries, the size of ovaries in PCOS patients is significantly greater (26). In addition, PCOS is strongly associated with metabolic disorders and hypothalamic–pituitary–ovarian axis function disorders and is characterized by multisystem reproductive metabolic disorders (27). Metabolomics has been shown to be a potential tool for researching the pathophysiology of PCOS. Rajska et al. conducted metabolomic research in individuals with PCOS and revealed that aberrant metabolic pathways are associated mainly with the metabolism of fatty acids, lipids, sphingolipids, glycerophospholipids, steroids, carbohydrates and amino acids (28). Despite its high incidence, the exact mechanism of PCOS is still unclear. Thus, it is necessary to identify metabolic markers involved in the pathophysiology of PCOS. The study of the gene expression of enzymes involved in metabolism is important for the early prognosis of complications and as a basis for clinical therapy.

POF is a common gynaecologic disease that can cause sterility, and it is characterized by low levels of oestrogen and high levels of follicle-stimulating hormone (FSH) prior to the age of 40 (29). Metabolic disorders such as autoimmune adrenal and thyroid diseases, chromosomal abnormalities, oxidative stress, diabetes, insulin resistance, and ovarian granulosa cell apoptosis are strongly related to the pathophysiology of POF (30, 31). Ngoh et al. reported that low expression of the mitochondrial membrane GTPase mitofusin 2 (Mfn2) promoted the apoptosis of granulosa cells and affected the synthesis of steroids by increasing stress in the endoplasmic reticulum (32). In addition, Chen et al. reported that low expression of Mfn2 in POF is associated with mitochondrial energy metabolism-related apoptosis in ovarian tissues (33). Thus, metabolic disorders promote the occurrence and progression of POF. Understanding aberrant metabolism provides new insight into clinical therapy.

Several studies have shown that BCAAs are metabolically aberrant in multiple diseases, even malignant cancers. OC, PCOS and POF are closely related to aberrant metabolism. Therefore, we reviewed the role of BCAA metabolism in OC, PCOS and POF and hope to provide new insights concerning clinical diagnosis and therapy.

## 2 BCAAs

**Figure 1:**
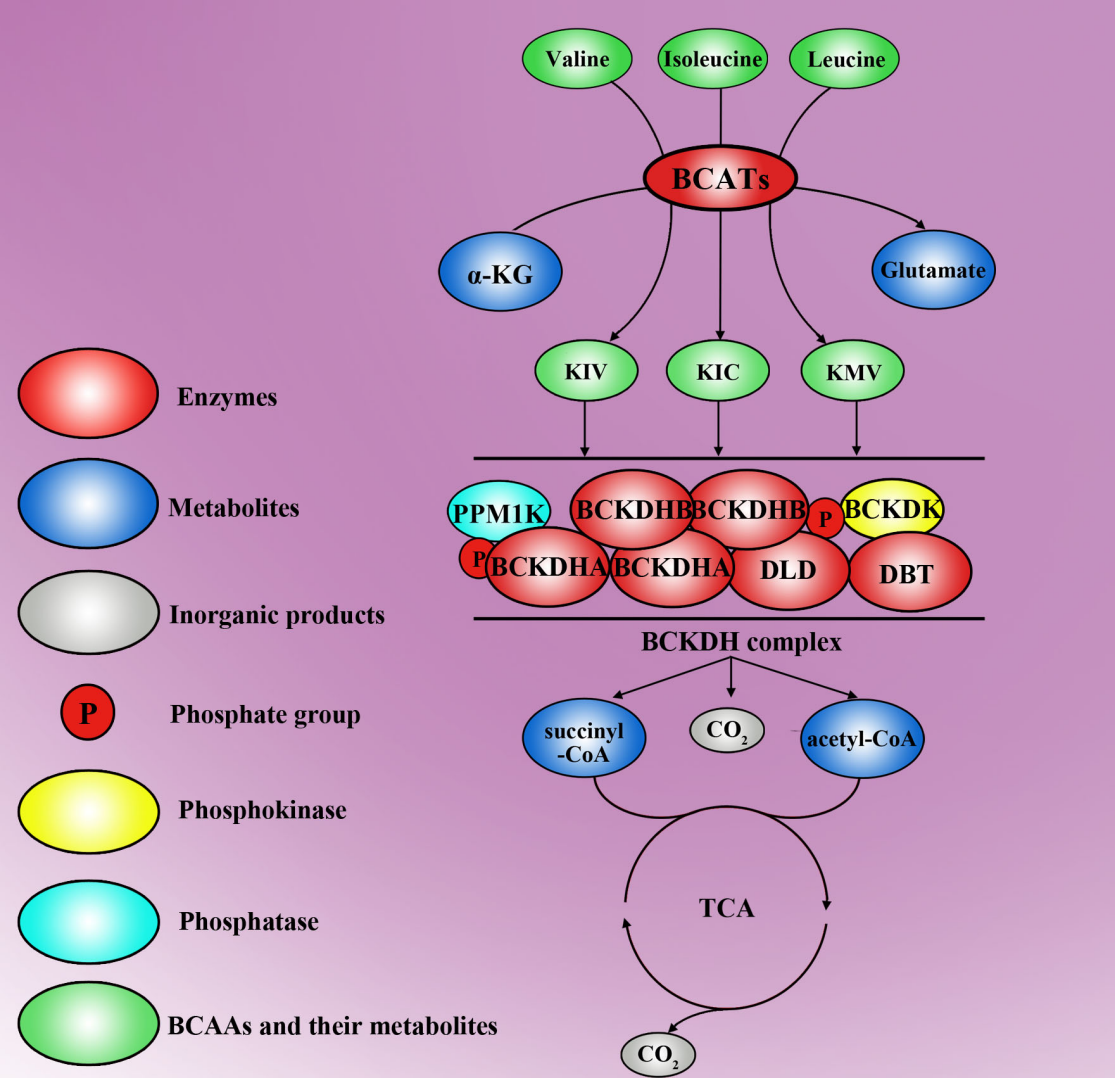
The molecular metabolic mechanisms of BCAAs. Valine, isoleucine and leucine are three types of BCAA, which are transformed into Kiv, Kic and Kmv, respectively, by the first key enzymes, BCATs. In this step, α-KG concurrently turns into glutamate. Moreover, Kiv, Kic and Kmv can be further decomposed into succinyl-CoA, acetyl-CoA and CO_2_ by the BCKDH complex. The BCKDH complex is another key enzyme for BCAA metabolism and is inactivated by PPM1K and BCKDK. Succinyl-CoA and acetyl-CoA can enter the tricarboxylic acid cycle and break down into H_2_O and CO_2_.

BCAAs are essential amino acids that are synthesized by algae, fungi, plants, and bacteria but not by animals. BCAAs include valine and leucine, which are carried out by the same enzymes, and isoleucine, which is created from α-ketoisovalerate and is the transamination precursor of valine (34). The catabolism of BCAAs can be roughly divided into two steps. BCATs are created by the first step of BCAA catalysis, in which BCAAs are converted to corresponding branched-chain α-keto acids (BCKAs), including α-ketoisocaproate, α-keto-β-methylvalerate, and α-ketoisovalerate, by transferring the amino group of BCAAs onto α-ketoglutaric acid (α-KG), thus producing glutamate (35). However, BCAT-catalysed transamination is reversible, which allows BCAAs to be generated by reamination of BCKAs from other tissues (36). α-KG is a key intermediate in the tricarboxylic acid (TCA) cycle, and glutamate is involved in the metabolism of several proteins, nucleotides and other nonessential amino acids (37, 38). BCKDH is created by the second step of BCAA catalysis, which is irreversible; in this step, oxidative decarboxylation is catalysed, thereby releasing CO_2_ and covalently adding a coenzyme A (CoA) group to the oxidized BCKAs, except for the valine catalysing pathway, which produces 3-hydroxyisobutyrate (3-HIB) (39). CoA is a bulky and hydrophilic prosthetic moiety that restricts all subsequent intermediates inside the mitochondria. BCA-CoA, including succinyl-CoA and acetyl-CoA, is catalysed by BCKDH and eventually enters the TCA cycle. There are three catalytic components in the BCKDH complex: branched chain α-ketoacid decarboxylase (E1), dihydrolipoyl transacylase (E2, encoded by the DBT gene) and dihydrolipoamide dehydrogenase (E3, encoded by the DLD gene). E1 is a thiamine-dependent enzyme that has two α subunits (E1α, encoded by the BCKDHA gene) and two β subunits (E1β, encoded by the BCKDHB gene) (40). When the level of BCAAs is low or when there is accumulation of BCAAS for protein synthesis, BCKDH becomes inactive. Protein phosphatase 2Cm (PP2Cm), a critical regulator of BCAA catabolism encoded by PPM1K, was found to be responsible for the dephosphorylation of BCKDH (41). However, BCKDK phosphorylates and inactivates the BCKDHA of this complex to inhibit the catabolism of BCAAs (42). BCAAs and their catabolic products act as signalling molecules that are involved in various functions of life. Aberrant BCAA metabolism leads to several diseases, and the metabolic production of BCAAs may be a potential predictor of disease severity (**Figure 1**).

## 3 Reviews on polycystic ovary syndrome and premature ovarian failure

### 3.1 BCAAs promote the development of polycystic ovary syndrome

First, studies have shown that the plasma levels of BCAAs are elevated in patients with PCOS (43, 44). Additionally, compared with normal pregnant women, PCOS patients exhibit differential expression of enzymes involved in BCAA metabolism during pregnancy, including upregulated BCKDHB and DBT and downregulated BCKDHA and BCKDK (45). The aberrant expression of these enzymes disrupts BCAA metabolism, leading to their accumulation in the blood.

**Figure 2:**
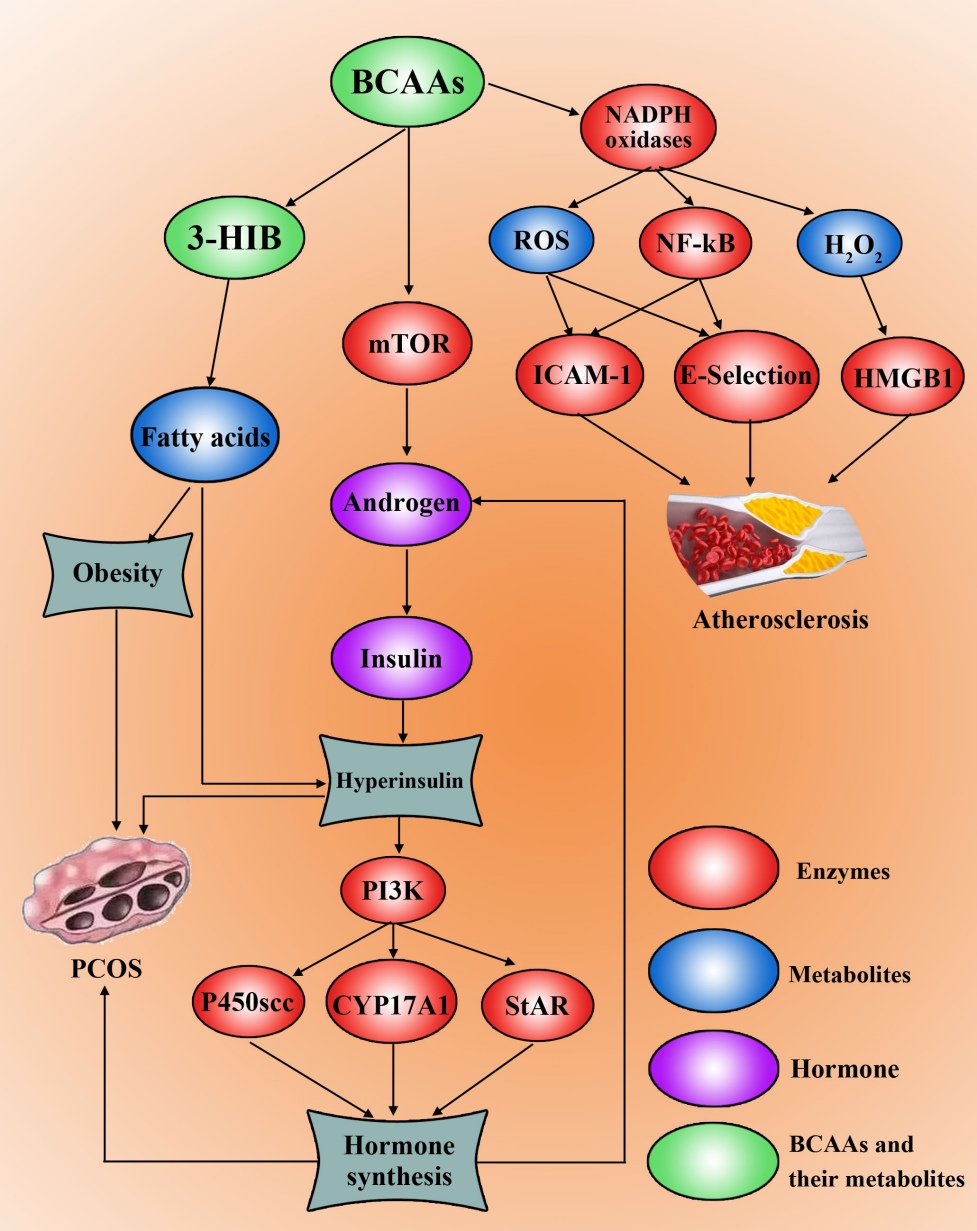
High levels of BCAAs promote PCOS development and progression. BCAAs can be metabolized to 3-HIB to synthesize fatty acids, leading to obesity. The amount of BCAAs can increase the level of androgen by activating mTOR, which induces insulin synthesis and hyperinsulinaemia and insulin resistance. Hyperinsulin can activate the PI3K pathway to increase P450scc, CYP17A1 and StAR to drive the synthesis of hormones, including androgens. Furthermore, BCAAs can induce the upregulation of NF-kB and the production of ROS and H_2_O_2_, which can induce the occurrence, development and progression of atherosclerosis in PCOS patients by ICAM-1, E-selectin and HMGB1.

Hyperinsulinaemia, hyperandrogenemia, and obesity collectively impair follicular development in PCOS patients. Interestingly, existing evidence suggests that abnormal BCAA metabolism may exacerbate insulin resistance, hyperandrogenaemia, and obesity (**Figure 2**). Jang et al. discovered that 3-HIB, a catabolic intermediate of the BCAA valine, promotes endothelial fatty acid transport, enhances fatty acid uptake, and induces lipid accumulation, ultimately causing insulin resistance in mice (46). Insulin resistance, in turn, worsens hyperandrogenaemia, further aggravating PCOS. Moreover, BCAAs participate in lipid metabolism, and elevated BCAA metabolic patterns correlate with increased serum triglyceride levels (47) and decreased high-density lipoprotein cholesterol levels (48), potentially exacerbating obesity and worsening hyperandrogenaemia in PCOS patients.

Although current research on the specific mechanisms linking BCAAs to PCOS remains limited, notably, BCAAs can positively regulate the mTOR signalling pathway through mechanisms governed on the lysosomal surface (49). Furthermore, Song et al. demonstrated that in a PCOS mouse model, leucine acts as a key agonist of mTOR, while activated mTOR causes mitochondrial dysfunction and reduced glucose uptake, thereby inducing insulin resistance (50). Concurrently, hyperactivation of mTORC1 increases androgen secretion (51). This suggests a pathological cascade: ​​aberrant enzymes involved in BCAA metabolism → BCAA accumulation in the blood → lysosomal activation of the mTOR signalling pathway → elevated androgen levels → insulin resistance and dyslipidaemia → ultimately leading to PCOS​​. Although this mechanism requires further investigation, it provides crucial insights into the pathogenesis of PCOS.

### 3.2 The functions of BCAAs in premature ovarian failure

Research on the role of BCAAs in POF suggests that BCAAs may compete with tyrosine, phenylalanine (a precursor of catecholamines), and tryptophan (a precursor of serotonin) for entry into the brain via the large neutral amino acid transporter (LNAA) (52). Reduced levels of catecholamines and serotonin could alter the synthesis and secretion of gonadotropin-releasing hormone (GnRH) in the hypothalamus (53). GnRH stimulates gonadotrophs to produce FSH and luteinizing hormone (LH), which are critical for ovulation and corpus luteum (CL) formation (54). Kang et al., using Illumina BeadChip technology for whole-genome genotyping, compared 16 POF patients (serum FSH >40 mIU/ml, age <40 years) with 16 perimenopausal women with regular menstrual cycles (age >40 years) and identified an association between the ​​BCKDHB​​ gene and POF (55).

Although the direct mechanisms linking BCAAs and POF remain unclear, several intriguing studies provide preliminary insights. Ernst et al. reported significant upregulation of mTOR signalling during the transition from human primordial follicles to primary follicles (56). Furthermore, Adhikari et al. reported that mice with a knockout of the mTOR suppressor gene ​​Tsc1​​ exhibited premature activation of the primordial follicle pool, leading to follicle depletion and POF (57). Similarly, Chen et al. demonstrated that cyclophosphamide (CTX), an alkylating agent, induced the apoptosis of growing follicles and primordial follicle loss in female mice via hyperactivation of the PI3K–Akt–mTOR pathway (58). Notably, the mechanisms found in mouse models, such as premature ovarian failure induced by CTX, may not be fully applicable to humans. Conversely, Alborzi et al. reported that leucine upregulated genes related to primordial follicle proliferation and differentiation (e.g., ​​Gdf9​​, ​​Bmf15​​) in murine ovarian tissue, although no changes in mTOR signalling were detected, suggesting that leucine may accelerate primordial follicle activation through nonmTOR pathways (59).

POF is characterized by the apoptosis of ovarian granulosa cells (60, 61). Jiang et al. proposed that elevated ROS inhibits telomerase reverse transcriptase (TERT) expression, thereby promoting POF (62). Furthermore, Zhenyukh et al. demonstrated that BCAAs (10 mmol/L) increased ROS production via NADPH oxidase and mitochondrial pathways (63), whereas Jiang et al. reported that BCAA treatment in rats caused myocardial injury through excessive ROS generation (64). These findings suggest that BCAAs may induce granulosa cell apoptosis via ROS-mediated mechanisms, contributing to POF, although the exact pathways involved require further investigation.

**Figure 3:**
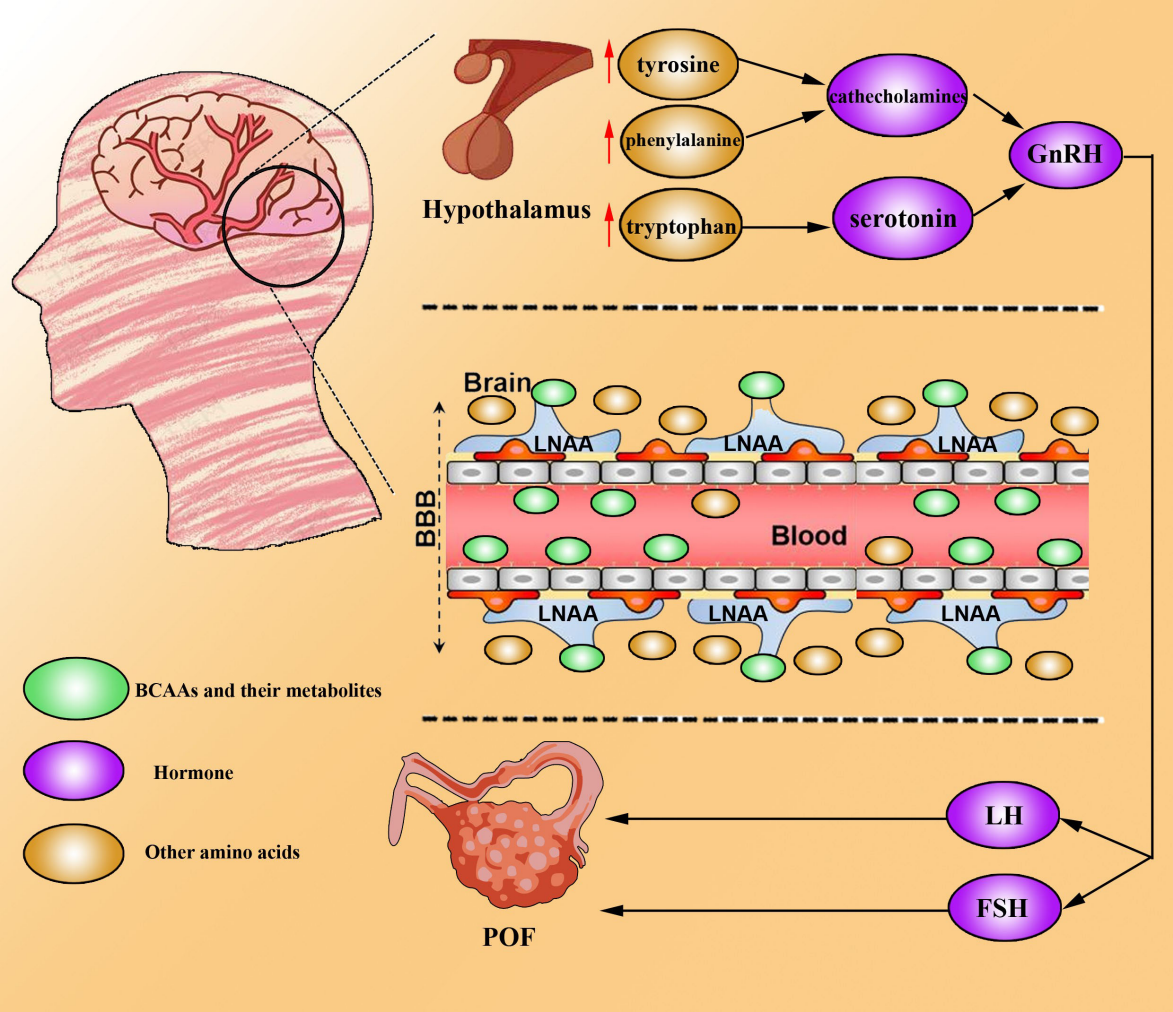
BCAAs drive POF development. BCAAs can competitively bind to LNAAs and be taken up by the brain across the blood–brain barrier. Hence, other amino acids (tyrosine, phenylalanine and tryptophan) accumulate in the brain, resulting in the upregulation of catecholamine and serotonin. High levels of catecholamines and serotonin increase the level of GnRH, which promotes the secretion of FSH and LH to induce the development and progression of POF.

Additional studies highlight potential links between BCAA metabolism, diabetes, and vascular dysfunction. Metabolomic analyses revealed strong associations between BCAAs and cardiovascular disease and insulin resistance (1). Mels et al. reported that elevated BCAAs in hyperglycaemic individuals correlate with worsened cardiovascular function (65). Notably, recent studies reported POF in ~2.5% of diabetic women (30). This raises the hypothesis that BCAA metabolism dysregulation may lead to diabetes or vascular complications, resulting in insufficient ovarian follicular blood supply and functional decline (**Figure 3**). Whether this axis contributes to POF pathogenesis warrants further exploration.

## 4 Association of the altered level of BBCAs and its metabolism towards ovarian cancers

Compared with normal cells, cancer cells are characterized by rapid proliferation and invasion of normal tissues, which require altered metabolism to meet increased nutritional and biosynthetic requirements (8, 66). However, an increasing number of studies have shown that BCAAs are important nutrients for cancer growth and are a source of energy that contribute to tumorigenesis (67), and OC is no exception. Several studies have shown that some enzymes involved in the first step of BCAA catalysis are overexpressed in multiple cancers (68–70). As BCAA metabolism increases, more BCA-CoA enters the tricarboxylic acid cycle. Metabolic enzymes of BCAAs include cytosolic BCAT1 and mitochondrial BCAT2 (71). BCAT2 is expressed in most tissues, while the expression of BCAT1 is restricted to the placenta, ovary and brain (35). BCAT1 has been reported to promote tumour proliferation, invasion, migration, cell cycle progression and apoptosis through multiple pathways (69, 72, 73).

Keita et al. used methylated DNA immunoprecipitation in combination with CpG island-tiling arrays and reported that BCAT1 is significantly hypomethylated in low-malignant potential serous epithelial ovarian cancer and high-grade serous epithelial ovarian cancer (74). Wang et al. reported that BCAT1 knockdown significantly inhibits proliferation, migration, invasion and S cell cycle arrest in EOC cells and A2780s cells and confirmed that BCAT1 suppression inhibits tumour metastasis and expansion in nude mice (75). Subsequently, Wang et al. using microarray assay confirmed that the difference gene after knockdown BCAT1 in SKOV3 involve in metabolism, regulation of transcription, transport, signal transduction, cell growth and cell cycle and using a kit-based high-throughput flow injection mass spectrometry approach confirmed that the suppression BCTA1 decrease major metabolite group including sphingolipids, glycerophospholipids and many amino acids, which could provide energy for tumour (75). Adipogenesis in normal tissues is restricted mainly to liver cells and adipocytes, whereas tumour cells can activate adipogenesis in response to their high metabolic needs (76). The overexpression of BCATs increases BCAA catalysis, resulting in the production of BCA-CoA, including succinyl-CoA and acetyl-CoA. Cytoplasmic acetyl-CoA is the main substrate for lipid synthesis. Both fatty acids and cholesterol are synthesized by acetyl-CoA, and their dysregulation is one of the most prominent metabolic changes in cancers. However, what factors influence the aberrant expression of BCATs? A previous study revealed that BCAT1 is a target gene of c-Myc genes in multiple cancers (68, 77, 78) and that the c-Mycxie’x gene, an oncogene, is overexpressed in EOC (79). As expected, Wang et al. confirmed that the knockdown of c-Myc suppresses the protein expression of BCAT1 (75). These findings suggest that the overexpression of c-Myc increases BCAT1 expression, resulting in the malignant development of EOC. In addition, several studies have reported that BCAT1 activates the mTOR pathway to contribute to tumorigenesis in many cancers (69, 80), although few studies have been reported in OC.

The BCAT reaction is reversible in response to changes in the concentrations of BCAAs and BCKAs. There are reports that the BCAA catabolism products BCA-CoA enter the tricarboxylic acid cycle to provide energy for tumorigenesis (81). However, other studies have shown that BCAA catabolism in tumour cells is decreased and that a high level of BCAT promotes the conversion of BCKAs to BCAA and α-KG and then provides essential nutrients and energy for tumorigenesis (82, 83). BCKDK, a negative regulatory enzyme in BCAA catabolism located in the mitochondrial matrix (84), is overexpressed in EOC and is strongly associated with the pathological grade of EOC patients (85). Li et al. used a Co-IP assay and reported that BCKDK interacts with MEK and suggested that BCKDK contributes to the proliferation and migration of OC cells SKOV3 and OVCAR3 by upregulating the MEK–ERK signalling pathway (85). Studies have shown that BCKDK combined with the overexpression of the phosphatase PPM1K, a ChREBP-regulated node, integrates BCAA metabolism and lipid metabolism via ATP-citrate lyase and promotes the use of BCAAs as materials for fat synthesis in fat cells, thereby providing energy for tumorigenesis (86). In addition, Zhai et al. reported that the phosphorylation of BCKDK may be mediated by aminopeptidase N (APN), which promotes the interaction of BCKDK with ERK1/2 to promote hepatocellular carcinoma proliferation and metastasis (87). In addition, Tian et al. suggested that the phosphorylation of BCKDK at Y246 by the nonreceptor protein tyrosine kinase Src improved the stability and activity of BCKDK and promoted invasion, migration and EMT in colorectal cancer cells (88). Thus, whether the phosphorylation of BCKDK by APN or Src also plays an essential role in OC needs further research.

**Figure 4:**
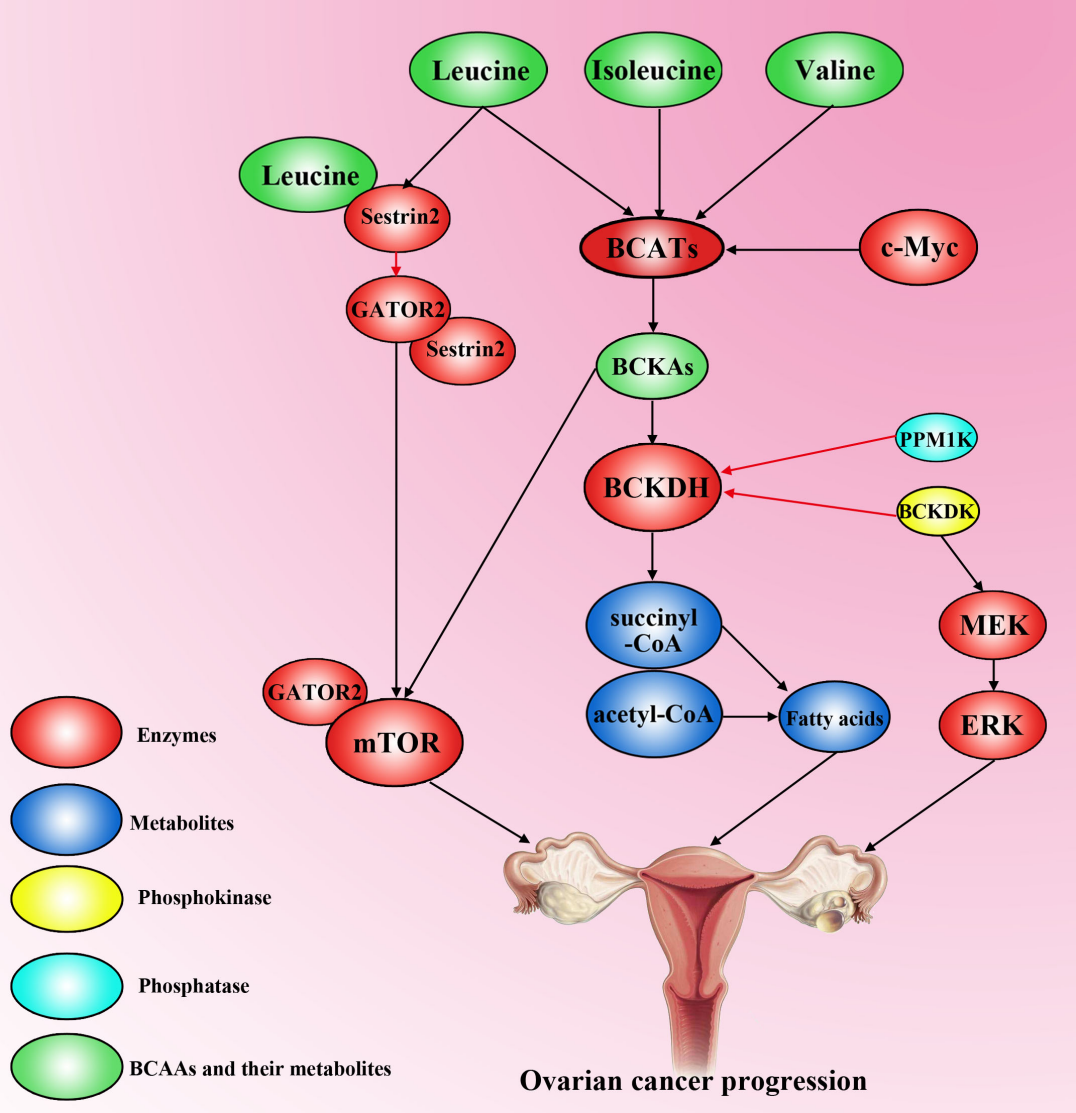
The effect of BCAAs on ovarian cancer progression. Valine, isoleucine and leucine are transformed into BCKAs (Kiv, Kic and Kmv) via BCATs, which can be activated by c-Myc. However, the activity of BCKDH is decreased by high levels of PPM1K and BCKDK in ovarian cancer cells. High accumulation of BCKAs activates mTOR to promote ovarian cancer progression. BCKDK overexpression can also activate the MEK–ERK pathway to accelerate the progression of ovarian cancer. Leucine can interact with sestrin2 to competitively inhibit the binding of sestrin2 and GATOR2, resulting in GATOR2 interacting with mTOR to promote tumorigenesis. Moreover, high levels of BCAAs increase fatty acid synthesis to accelerate ovarian cancer development.

The overexpression of BCKDK inhibits the conversion of BCKAs to BCA-CoA, resulting in the accumulation of BCKAs, and the accumulation of BCKAs inhibits BCAA catabolism (85). Several studies have shown that leucine (not valine, isoleucine, or BCKAs) is a well-described mTOR agonist that activates mTORC1 by directly interacting with Sestrin2, a negative regulator of mTORC1 activity, and Sestrin2 directly inhibits GATOR2, a positive regulator of mTORC1, in the absence of leucine (89). However, mTOR is involved in the regulation of autophagy, apoptosis, and tumour proliferation via multiple signalling pathways in OC (90, 91). Whether the accumulation of BCAAs activates the mTOR pathway to contribute to the development of OC needs further study (**Figure 4**).

## 5 Conclusion

BCAAs are the basis for all life forms. Recent studies have suggested that BCAAs may play an essential role in the development of troubling worldwide epidemic diseases. Ovarian diseases afflict many women; examples include OC, which has a high mortality rate and poor prognosis, and PCOS and POF, which afflict women with infertility and lead to serious complications such as insulin resistance, diabetes, and obesity. However, it is still controversial whether metabolic disorders are a cause or a result of disease. According to the current data, metabolic disorders may aggravate the occurrence and development of this disease.

In ovarian cancer patients, aberrant BCAA metabolism may promote tumour proliferation, migration and invasion. Research findings suggest that the use of BCKDK inhibitors (BCKDKis) can rescue ovarian cancer resistance by reducing BCAA levels (92). However, while BCKDK is highly expressed in various ovarian cancer cell lines compared with normal ovarian epithelial cells, its expression is significantly lower in the COV-318 and OVCAR-3 cell lines (92). This variability indicates that relying on BCKDK as a diagnostic or prognostic marker may yield false-negative results. Additionally, inhibiting BCAT1 expression in ovarian cancer can reduce amino acid levels and significantly suppress tumour progression both *in vitro* and *in vivo* (75). Nevertheless, BCAT1 may serve as a more reliable diagnostic and prognostic marker for ovarian cancer. Studies have shown that BCAT1 levels are markedly elevated in the blood and ovarian tumour tissues of ovarian cancer patients and that its overexpression in tumour tissues is closely associated with chemotherapy resistance (93–95). For patients with PCOS, abnormal BCAA metabolism may promote insulin resistance, obesity, and hyperandrogenaemia. However, a clinical study of PCOS samples revealed no significant differences in BCAT2 expression within the BCAA metabolic pathway, whereas BCKDK expression was notably reduced. These findings suggest that relying solely on BCKDK as a diagnostic marker may lack reliability, highlighting the need to further elucidate the specific mechanisms of BCAAs in PCOS (45). The level of BCAAs in the serum of patients with premature ovarian failure is high, which may lead to POF by reducing the expression of FSH and LH and the degree of corpus luteum formation. However, whether BCAT1 and BCKDK can be used as diagnostic markers of POF still needs further mechanistic discussion and research (**Table 1**).

**Table 1:** The functions of BCAA related enzymes in ovarian diseases.

Disease name	BCAA related enzymes	Expression	Function
OC	BCAT1	up	promote
OC	BCAT2	/	/
OC	BCKDHA	up	promote
OC	BCKDHB	up	promote
OC	BCKDK	up	promote
PCOS	BCAT1	/	promote
PCOS	BCAT2	/	/
PCOS	BCKDHA	down	/
PCOS	BCKDHB	up	/
PCOS	BCKDK	down	/
POF	BCAT1	/	/
POF	BCAT2	/	/
POF	BCKDHA	/	/
POF	BCKDHB	up	promote
POF	BCKDK	/	/

Currently, many people use BCAAs as an energy supplement to build muscle. However, the negative factors associated with BCAAs cannot be ignored. For patients with tumours, supplementation with BCAAs may promote tumorigenesis. The level of BCAAs is elevated in PCOS and POF patients, and supplementation with BCAAs may aggravate metabolic disorders and lead to disease deterioration. For patients with metabolic disease, BCAA supplementation after exercise is not recommended. We reviewed BCAA metabolism in OC, PCOS and POF, hoping to provide suggestions for future clinical diagnosis or management.
